# Association of Anti-Mullerian Hormone with C-Reactive Protein in Men

**DOI:** 10.1038/s41598-019-49596-x

**Published:** 2019-09-11

**Authors:** Dinesh Kadariya, Nargiza Kurbanova, Rehan Qayyum

**Affiliations:** Department of Internal Medicine, Virginia Commonwealth University Medical Center, Richmond, Virginia USA

**Keywords:** Predictive markers, Risk factors

## Abstract

While serum anti-mullerian hormone (AMH) levels are inversely associated with all-cause mortality in men, the underlying mechanisms are unclear. Elevated levels of inflammation, also associated with all-cause mortality, and may be the link between AMH and mortality. Hence, we examined the association of AMH with serum c-reactive protein (CRP), a biomarker of inflammation, in men. We included men ≥20 years from the National Health and Nutrition Examination Survey (1999–2004). We used survey weight-adjusted linear regression to examine the association between AMH and CRP without and with adjustment for age, race, body mass index (BMI), smoking, hypertension, diabetes, cholesterol, glomerular filtration rate (GFR), testosterone, androstenedione, and sex hormone binding globulin. Of the 949 men, 212 (22%) were elderly, 493 (52%) Caucasian, 254 (27%) current smokers, 100 (10%) diabetics, and 312 (33%) had hypertension. Mean (SD) AMH was 8.4 (7.2) ng/mL and median (IQR) CRP level was 0.17 (3) mg/L. Using linear regression, each 10 ng/mL rise in AMH was associated with 0.09 mg/dL (95%CI = −0.14 to −0.03; p = 0.002) decrease before and 0.08 mg/dL (95%CI = −0.13 to −0.02; p = 0.004) decrease in CRP after adjusting for potential confounders. Similarly, men in the highest quartile of AMH had significantly lower CRP compared to those in the lowest quartile (unadjusted difference = −0.19 mg/dL; 95%CI = −0.31 to −0.06; p = 0.006, adjusted difference = −0.16 mg/dL; 95%CI = −0.3 to −0.01; p = 0.035). We found an independent, robust, and inverse association between CRP and AMH in men. Effect of AMH on mortality may be through amelioration of inflammation.

## Introduction

Anti-mullerian hormone (AMH), is a glycoprotein belonging to the transforming growth factor-beta (TGF-β) superfamily. It is highly expressed in male fetal testis where it is responsible for regression of the mullerian duct. AMH remains elevated until puberty in men and rapidly declines during transition to the adulthood^1,2^. While the role of the persistence of AMH during adulthood is unclear, the presence of AMH-specific receptor (AMHRII) in several organs (such as adrenal gland, liver, lung, skeletal muscle, and spleen) suggests that AMH may play a role during adult life beyond the development of reproductive system^3^. In fact, epidemiological studies have demonstrated that serum AMH levels were inversely associated with cardiovascular events in elderly male, with infra-renal aortic diameter, and with all-cause mortality in men^4–6^. However, the mechanisms underlying of these associations are not known.

Growing body of evidence suggests that inflammation plays a critical role in the initiation and progression of atherosclerosis, chronic pulmonary disease (including COPD), and malignancy^7–9^. Several studies have reported that serum c-reactive protein (CRP), an inflammatory biomarker, has the ability to predict cardiovascular disease, lung disease, cancer, and all-cause mortality risk in general population^10,11^. In one study, serum AMH levels were negatively associated with interleukin-6 level, an inflammatory cytokine, in men^12^. Therefore, we hypothesized that AMH and inflammation, and hence CRP, may be inversely associated with one another based on their proposed effects on clinical outcomes and aimed to examine this association between AMH and CRP in a large cohort of men.

## Methods

We used the publicly-available National Health and Nutrition Examination Surveys (NHANES) data to explore the relationship between AMH and CRP levels. NHANES is a complex, multistage, probability sampling series of surveys of the noninstitutionalized United States population with a goal to determine population’s health and nutritional status. The surveys oversample participants from certain population subgroups, such as African-Americans and Mexican-Americans, to ensure precision and reliability of estimates. Detailed methods of surveys are available on the NHANES website (http://www.cdc.gov/nchs/nhanes.htm). All participants provided informed consent and National Center for Health Statistics Research Ethics Review Board approved all protocols.

The three NHANES continuous cycles (1999–2000, 2001–2002, 2003–2004), which were included in our study, had serum AMH levels measured in approximately 1/3 subsample of men. These men were selected based on availability of stored samples. Individuals <20 years of age or with missing AMH or CRP data were excluded. Data on age, sex, and gender were collected through self-report by study participants. Participants were subcategorized into three age groups (young =≤ 35 years, middle-age => 35 & <65 years, and elderly =≥ 65 years). We categorized participants as smokers if they were actively smoking at baseline, ever smokers if they had ever smoked ≥100 cigarettes during their lifetime, and nonsmokers if they had never smoked. We calculated BMI by dividing body weight (in kilograms) with height (in meters squared). We categorized participants as hypertensives if their systolic blood pressure was >140 mm Hg, if their diastolic blood pressure was >90 mm Hg, if they had history of hypertension, or if they were taking antihypertensive medications. We inferred the presence of diabetes mellitus if participants had a prior diagnosis of diabetes mellitus, were taking insulin or other oral medication for the blood glucose management, had hemoglobin A1C >7.0%, or had a blood glucose level >200 mg/dl.

Serum AMH levels were measured on frozen stored samples using AMH Gen II ELISA (Beckman Coulter, USA) apparatus that has coefficient of variation of <10% and uses an enzymatically amplified two-site immunoassay^13^. Serum CRP levels were measured using nephlometer (Behring Nephelometer, Germany). Serum creatinine levels were measured using the Jaffe rate method (kinetic alkaline picrate). We estimated glomerular filtration rate (GFR) using Chronic Kidney Disease Epidemiology Collaboration equation^14^. Serum glucose was measured using Beckman Synchron LX20 (Beckman Coulter, Brea, Calif) and hemoglobin A1C was measured using the Primus CLC300 (Primus Corporation, Kansas City, MO). Sex hormone binding globulin (SHBG), estradiol, and total testosterone were measured using immunoassays based on emitted chemiluminescence. Androstenedione was measured by first, mixing patient samples containing unlabeled antigen with enzyme labeled antigen then, removing unbound materials and finally, measuring absorbance.

All analyses were performed using appropriate adjustments to survey weights for the 3- cycles of the NHANES. The sampling weights reflect the complex survey sampling method and were assigned to each person to account for the unequal probability of selection and nonresponse. In analyses, serum AMH was included as continuous variable and after dividing into quartiles. Relationships between categorical variables were calculated using Pearson’s Chi-square test. Relationships between continuous variables and AMH quartiles were examined using Student’s t-test, analysis of variance (ANOVA) or Kruskal-Wallis’ rank test as appropriate. Survey weight-adjusted linear regression was conducted to examine the association of AMH with CRP without and with adjustment for potential confounders. We used a *p*-value of <0.05 as statistically significant. Multiple linear regression analyses were adjusted for age, race, BMI, hypertension, diabetes, smoking status, total cholesterol, estimated GFR, serum total testosterone, SHBG, and androstenedione. All analyses were performed on Stata/MP version 14.1 (Stata Corp LP, College Station, Texas).

### Ethical approval

All procedures performed in studies involving human participants were in accordance with the ethical standards of the institutional and/or national research committee and with the 1964 Helsinki declaration and its later amendments or comparable ethical standards.

### Informed consent

Written informed consent was taken from each participant for both the in-home interview and the health exam during the survey.

## Results

Of the 949 men, 212 (22%) were elderly, 493 (52%) were Caucasian, 254 (27%) current smokers, 100 (10%) were diabetics, and 312 (33%) had hypertension. Mean (SD) AMH was 8.4 (7.2) ng/mL and median (IQR) CRP level was 0.17 (3) mg/dL. When comparing the lowest quartile of AMH (1.9 [1.1] ng/mL) with the highest quartile (18.6[7.6] ng/mL), the later had more young men, lower BMI, and fewer Caucasians, diabetics, and hypertensives (all P < 0.001; Table 1). Elderly participants had higher CRP levels (geometric mean: 0.22 mg/dL) compared to middle-age and young participants (geometric mean: 0.21 and 0.13 mg/dL, respectively, all *p*-values < 0.001). There was no statistically significant difference in CRP levels between Caucasians and non-Caucasians (geometric mean: 0.17 vs. 0.17 mg/dL; p = 0.78). Serum CRP levels were higher in current smokers than in ever-smokers or non-smokers (geometric means: 0.20, 0.18 and 0.14 mg/dL, respectively; p = 0.001), in diabetics than in non-diabetics (geometric mean: 0.3 vs. 0.2 mg/dL; p < 0.001), and in hypertensive than in normotensive participants (geometric mean: 0.22 vs. 0.15 mg/dL; p < 0.001). We observed an inverse and significant correlation between serum CRP and AMH levels (r = −0.07; p = 0.02). While CRP had an inverse and statistically significant relationship with serum testosterone (r = −0.12; p < 0.001), there was no correlation between CRP and androstenedione (r = 0.01; p = 0.8) or SHBG (r = −0.06; p = 0.07).

**Table 1:** Study population characteristics by anti-mullerian hormone quartiles.

Variable	Q1 (N = 238)	Q2 (N = 237)	Q3 (N = 237)	Q4 (N = 237)	P-value
Age, years, *mean (SD*)	57.9 (17.8)	51.6 (16.9)	45 (18)	40.5 (16.1)	<0.001
**Age** (**in categories)**					
Young, *n* (*%*)	63 (26.5)	88 (37.1)	135 (67.5)	160 (67.5)	
Middle Age, *n* (*%*)	82 (34.45)	96 (40.5)	58 (24.5)	55 (23.2)	<0.001
Elderly, *n* (*%*)	93 (39.1)	53 (22.4)	44 (18.6)	22 (9.3)	
**Race**					
Caucasians, *n* (*%*)	144 (60.5)	132 (55.7)	129 (54.4)	88 (37.1)	<0.001
CRP, mg/dL, *median* (*IQR*)	0.23 (0.39)	0.2 (0.27)	0.15 (0.3)	0.12 (0.21)	<0.001
AMH, ng/mL, *mean* (*SD*)	1.9 (1.1)	4.9 (0.8)	8.2 (1.3)	18.6 (7.6)	<0.001
Diabetes, *n* (*%*)	40 (16.8)	24 (10.1)	20 (8.4)	16 (6.75)	0.002
Hypertension, *n* (*%*)	107 (45)	81 (34.2)	66 (27.8)	58 (24.5)	<0.001
BMI, kg/m^2^, *mean* (*SD*)	28.8 (6.1)	28.1 (5.3)	27.7 (5.3)	26.5 (4.7)	<0.001
**Smoking**					
Current smokers, n (*%*)	64 (26.9)	61 (25.8)	52 (21.9)	77 (32.5)	0.002
Ever smokers, *n* (*%*)	85 (35.7)	80 (33.9)	75 (31.6)	47 (19.8)	
Non-smokers, *n* (*%*)	89 (37.4)	95 (40.25)	110 (46.4)	113 (47.7)	
GFR, mL/min, *mean* (*SD*)	84.9 (23.6)	90 (20.7)	96 (20)	99 (19.3)	<0.001
Cholesterol, mg/dL*, mean* (*SD*)	198.3 (53.3)	197.9 (40.7)	198 (34.9)	194.2 (40.3)	0.69
Androstenedione, ng/mL, *mean* (*SD*)	8.7 (7.9)	8.04 (4.95)	7.6 (3.9)	8.01 (4.7)	0.25
Testosterone, ng/mL, *mean* (*SD*)	4.7 (3.6)	5.15 (2.3)	5 (2)	5.55 (2.2)	0.005
SHBG, nmol/L, *mean* (*SD*)	45 (24.3)	44.7 (30.2)	38.1 (21.3)	38.2 (24.6)	0.001

^*^CRP = c - reactive protein; AMH = anti-mullerian hormone; BMI = body mass index; GFR = glomerular filtration rate (calculated using CKD-EPI (chronic kidney disease epidemiology collaboration equation); SHBG = sex hormone-binding globulin.

^**^P-values for categorical variables were calculated using Pearson’s Chi-square test; P-values for continuous variables were calculated using analysis of variance (ANOVA) and for CRP Kruskal-Wallis’ rank test.

**Figure 1:**
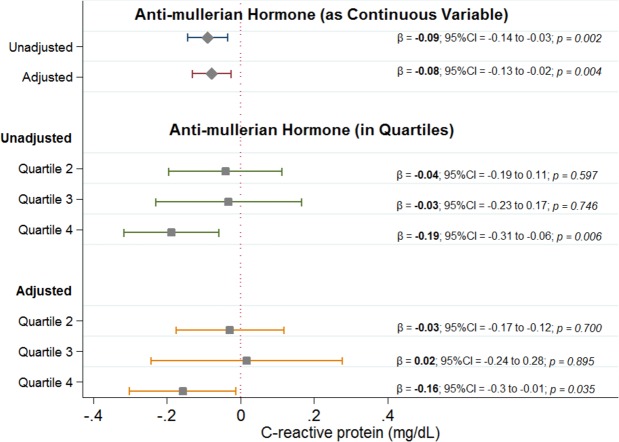
Results of unadjusted and adjusted survey weight-adjusted linear regression analyses. The first quartile is a reference hence not shown in the figure.

**Figure 2:**
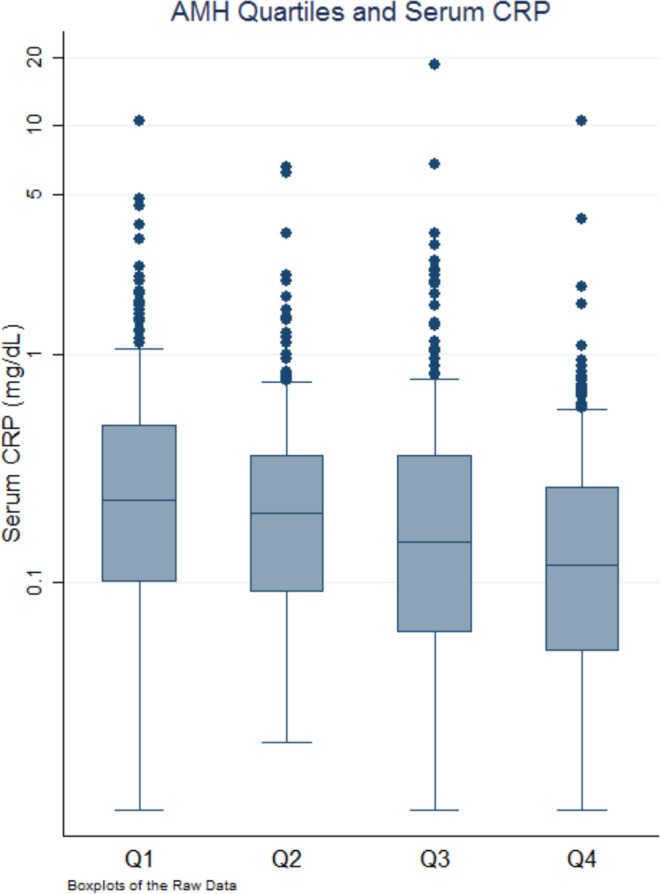
Association of c-reactive protein (CRP) with anti-mullerian hormone (AMH) divided into quartiles. CRP level decreases as AMH level increases from 1^st^ quartile to 4^th^ quartile.

In unadjusted linear regression, each 10 ng/mL increase in AMH was associated with 0.09 mg/dL decrease in CRP (95%CI = −0.14 to −0.03; p = 0.002). The results were robust to adjustment for potential confounding variables; each 10 ng/mL increase in AMH was associated with a 0.08 mg/dL decrease in serum CRP levels (95%CI = −0.13 to −0.02; p = 0.004) **(**Fig. 1). When examined by quartiles of AMH, men in the highest quartile of AMH had significantly lower CRP levels compared to men in the lowest quartile (−0.19 mg/dL; 95%CI = −0.31 to −0.06; p = 0.006) (Fig. 2) and this difference remained significant after adjusting for potential confounding variables (−0.16 mg/dL; 95%CI = −0.3 to −0.01; p = 0.035).

## Discussion

We report an inverse relationship between serum AMH levels and CRP levels in this large cohort of men representative of the United States adult population. The observed relationship was robust and independent of potential confounders such as age, race, smoking status, BMI, hypertension, diabetes, estimated GFR, total cholesterol, total testosterone, SHBG, and androstenedione. Further, the relationship was independent of the distribution of AMH and was seen even when examined with quartiles of AMH. To our knowledge, this is the first study to show this relationship although the exact mechanism of this association needs further exploration.

AMH belongs to the transforming growth factor-beta (TGF-β) superfamily that includes TGF-β and bone morphogenetic proteins (BMPs). It is secreted as proAMH and is cleaved by enzymes found in vascular tissues^15,16^. Once cleaved, it binds and activates non-specific receptors (AMH type I or activin receptor-like protein kinases [ALK2, ALK3, or ALK6]) or specific receptor (AMHRII). Activated receptors phosphorylate SMAD cytoplasmic proteins which translocate to the nucleus and modulate gene expression^17^.

AMH plays an important biological role in embryological development of male fetus by inducing the regression of mullerian ducts^18^. AMH levels remain high in male until puberty when its levels decline. These levels are negatively regulated by serum testosterone levels and are upregulated by follicular stimulating hormone^19^. Due to the central role of Sertoli cells in maintaining its levels, AMH has been proposed as a marker of Sertoli cell and testicular function. Further, it’s serum levels may be used to distinguish between anorchia and bilateral cryptorchidism, between testicular dysgenesis and dissociated tubular interstitial dysfunction, and between persistent mullerian duct syndrome and other intersex states^19,20^.

Several lines of evidence from molecular, cellular, animal, and epidemiological studies suggests biological plausibility for this association. Although its function in cardiovascular tissues remains unclear, the presence of AMHRII in heart and vasculature and proAMH-cleaving enzymes in vascular tissues indicate that cardiovascular system may be a putative target for AMH^21–23^. AMH has been shown to induce the expression of SMAD6 and, to a lesser extent of, SMAD7 in mouse Leydig cells and may do so in other cells; both SMAD6 and SMAD7 are important modulators of inflammation through regulation of BMP and TGF-beta/activin signaling^24,25^. In animal studies, exposure of chronic inflammation in pregnant rats using intraperitoneal lipopolysaccharide injections resulted in intrauterine growth restriction and was associated with significantly lower levels of serum AMH in offspring^26^. In female monkey (Cynomolgus macaques) model, serum AMH concentration was inversely related to the risk of atherosclerosis^27^.

Several epidemiological studies have explored the role of serum AMH as a marker of ovarian reserve and fertility in women but only a few studies have demonstrated its inverse association with atherosclerosis and cardiovascular disease^28–30^. Even fewer studies examining the role of AMH with inflammation have been conducted in men. In women, lower AMH was associated with higher serum CRP levels^31^. Further, lower AMH levels were also found in women with Crohn’s disease, with polymyositis, and in those with juvenile idiopathic arthritis as compared to healthy controls^32–34^. Serum AMH levels are also negatively associated with interleukin-6 levels in men^12^. Abundant data suggest that inflammation is involved in the atherosclerosis, lung disease, and malignancy and that anti-inflammatory therapy reduces the incidence of cardiovascular events and lung cancer in high-risk patients^35,36^. Thus, AMH, through its effect on inflammation, may reduce cardiovascular disease, COPD, and malignancy and hence overall mortality. However, further studies are needed to strengthen the plausibility for a causal argument of an inverse relationship between AMH and inflammation, morbidity, and mortality.

Our study has important research implications. While AMH is not currently thought to play a vital role during adult life, its inverse association with mortality and with CRP, an inflammatory biomarker, suggests further research on AMH’s role in human disease, mortality, and perhaps longevity is needed. This includes research in better understanding of the genetic and environmental determinants of the AMH levels, molecular signaling pathways, and its interactome. Further, as there is an ongoing research in developing AMH analogues, understanding and examining the effect of any such analogues on non-reproductive systems will also be important^37^.

Our study has several strengths and potential limitations. We used data representative of the adult men in the United States population. Our results are robust to underlying statistical assumptions about the AMH distribution. As with any observational study, association found in this study does not prove causality. Examining the effect of serum FSH on the relationship between serum CRP and AMH would have been important, however, serum FSH levels were not measured in men who had AMH levels measured. Another potential limitation is that serum CRP levels and AMH levels were measured once and, hence, inferring a longitudinal relationship is not possible with some assumptions. In fact, AMH has been shown to be variable on repeat sampling in women^38^. However, such variability should result in bias of effect estimates towards null and the true effect size of the relationship may be larger than what is observed in our study.

In summary, results from our study demonstrate a robust inverse correlation of AMH with CRP levels. Although cross-sectional, the findings of the present study call for additional studies to test the hypothesis that modulation of inflammatory processes is a potential pathway by which AMH reduces all-cause mortality in men.
